# Incorporating Real World Experiences in an Undergraduate Biochemistry Laboratory Course—Detection of Gluten in Food and Beverages Using an Enzyme-Linked Immunosorbent Assay

**DOI:** 10.1021/acsomega.5c07479

**Published:** 2025-09-30

**Authors:** Reika Haskell, Tanea T. Reed

**Affiliations:** 6885 Eastern Kentucky University, 521 Lancaster Avenue, Richmond, Kentucky 40475, United States

## Abstract

Celiac disease (CD), an autoimmune disease affecting 1% of the global population, is caused by the consumption of gluten. Gluten is a storage protein that is found in wheat, rye, and barley. The addition of gluten to food products imparts unique viscous and elastic characteristics in foods such as bread. The only recommended treatment for celiac disease is a gluten-free diet. The consumption of gluten-free labeled food products reduces the risk of an autoimmune response and other symptoms associated with celiac disease, including anemia, dermatitis herpetiformia, osteoporosis, muscle weakness, ataxia, and coagulopathy. The Codex Alimentarius and Food and Drug Administration (FDA) state that gluten-free foods must limit gluten content to 20 ppm. Using an enzyme-linked immunosorbent assay (ELISA), quantitative studies can be performed to detect the concentration of gluten present in gluten-free food products. This work uses a direct ELISA to quantify the amount of antigen (gluten) present by measuring absorbance in gluten-containing and gluten-free food products. A color gradient was observed based on the increasing concentration of gluten present in each gluten standard. Multiple substrates (*o*-phenylenediamine, 3,3′,5′-tetramethylbenzidene, aminosalicylic acid, and 2,2′-azino-bis­(3-ethylbenzthiazoline-6-sulfonic-acid)) were used to determine the most efficient method for incorporating this technology in an undergraduate biochemistry laboratory experiment where time is restricted and resources may be limited. This experiment was optimized to complete this assay within a 2.5 h lab period, thereby demonstrating a real world application using traditional biochemistry techniques and enhancing the students’ laboratory skill set and critical thinking skills.

## Graphical abstract

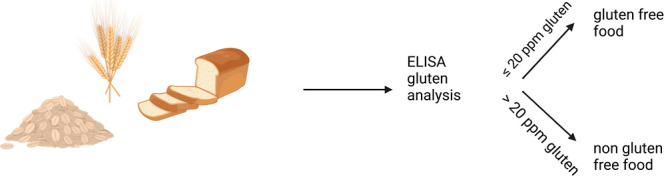

## Introduction

Gluten is a water-insoluble storage protein found in wheat, rye, and barley. Prolamins and glutelins are the two main classifications of gluten. Prolamins are alcohol-soluble, monomeric proteins, while glutelins are alcohol-insoluble, polymeric proteins. The addition of gluten to food products imparts unique viscous and elastic characteristics to foods such as bread.^1^ Gliadin, a prolamin, is the major component of gluten and is a target of study. Celiac disease (CD), an autoimmune disease affecting 1% of the global population, is caused by the consumption of gluten. Most cases of celiac disease are undiagnosed; however, those who have Type 1 diabetes and are first degree relatives of persons with CD have a greater incidence of having the disease.^2^ The only recommended treatment for celiac disease is a gluten-free diet; therefore, individuals with CD must refrain from consuming foods that contain gluten. The consumption of gluten-free labeled food products reduces the risk of an autoimmune response and other symptoms associated with celiac disease, including anemia, dermatitis herpetiformia, osteoporosis, muscle weakness, ataxia, and coagulopathy. According to the United States Food and Drug Administration (FDA), gluten-free labeled food can have a maximum of 20 ppm of gluten.^3^ Using an enzyme-linked immunosorbent assay (ELISA), quantitative studies can be performed to quantify the gluten present in gluten-containing and gluten-free food products, thereby providing consumers with more knowledge in making food choices, especially those with celiac disease. The resultant color of the ELISA reaction is proportional to the amount of antigen present in the wells. The general peroxidase reaction is shown in Figure 1.

**Figure 1:**
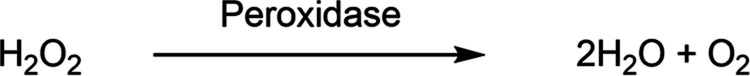
General peroxidase reaction.

This work established and optimized a protocol using direct ELISA to quantify the amount of antigen (gliadin) present by measuring the absorbance. The protocol used was modified from Iametti^3^ and Mena.^4^ Qualitative observations based on color intensity for each well were completed. Samples with less gluten (i.e., gluten-free foods) were expected to demonstrate a less intense color. The goal of this work was to optimize an undergraduate laboratory experiment using this traditional biochemical technique that could be completed by undergraduates within one laboratory session (<2.5 h). Students would be able to evaluate products containing gluten and several that are labeled gluten-free to determine if the labeling is accurate based on FDA regulations. In order to optimize the results, a total of four modifications were established. The concentration of gluten in the standards was increased, producing a more linear standard curve and a visible color gradient. Incubation times were reduced in order to decrease the total time from approximately 5 to 1.5 h to ensure this experiment could be completed within a 2.5 h time block. Based on the results, reducing the incubation time had minimal effects on reproducibility. Gluten was extracted from food samples using 60% ethanol, with expected results of foods that are gluten-free having lower absorbance measurements than gluten-containing foods. The extraction of gluten using 60% ethanol ensures the antibody is only interacting with prolamins present in the food products. Additional extraction methods using a mixture of guanidine hydrochloride and 2-mercaptoethanol, as well as the patented Universal gluten extraction solution (an arginine-based hydroalcohol), have shown promise, but were not used due to safety concerns and expense.^5^ Lastly, four different substrates were used to observe both the color present and the absorbance measurements every 5 min for a total of 30 min. Four substrates (*o*-phenylenediamine, 3,3′,5′-tetramethylbenzidene, aminosalicylic acid, and 2,2′-azino-bis­(3-ethylbenzthiazoline-6-sulfonic-acid)) were used to determine the most efficient method for incorporating this technology in an undergraduate biochemistry laboratory experiment where time is restricted and resources may be limited. A list of their properties can be found in Table 1. From the modifications listed, the best conditions to be used in future experiments are as follows: freshly prepared (<1 h before use) gluten standards, freshly prepared (<1 h before use) gluten extracts from food samples using 60% ethanol (v/v), and using the *o*-phenylenediamine substrate after 25 min of incubation.

**Table 1:**
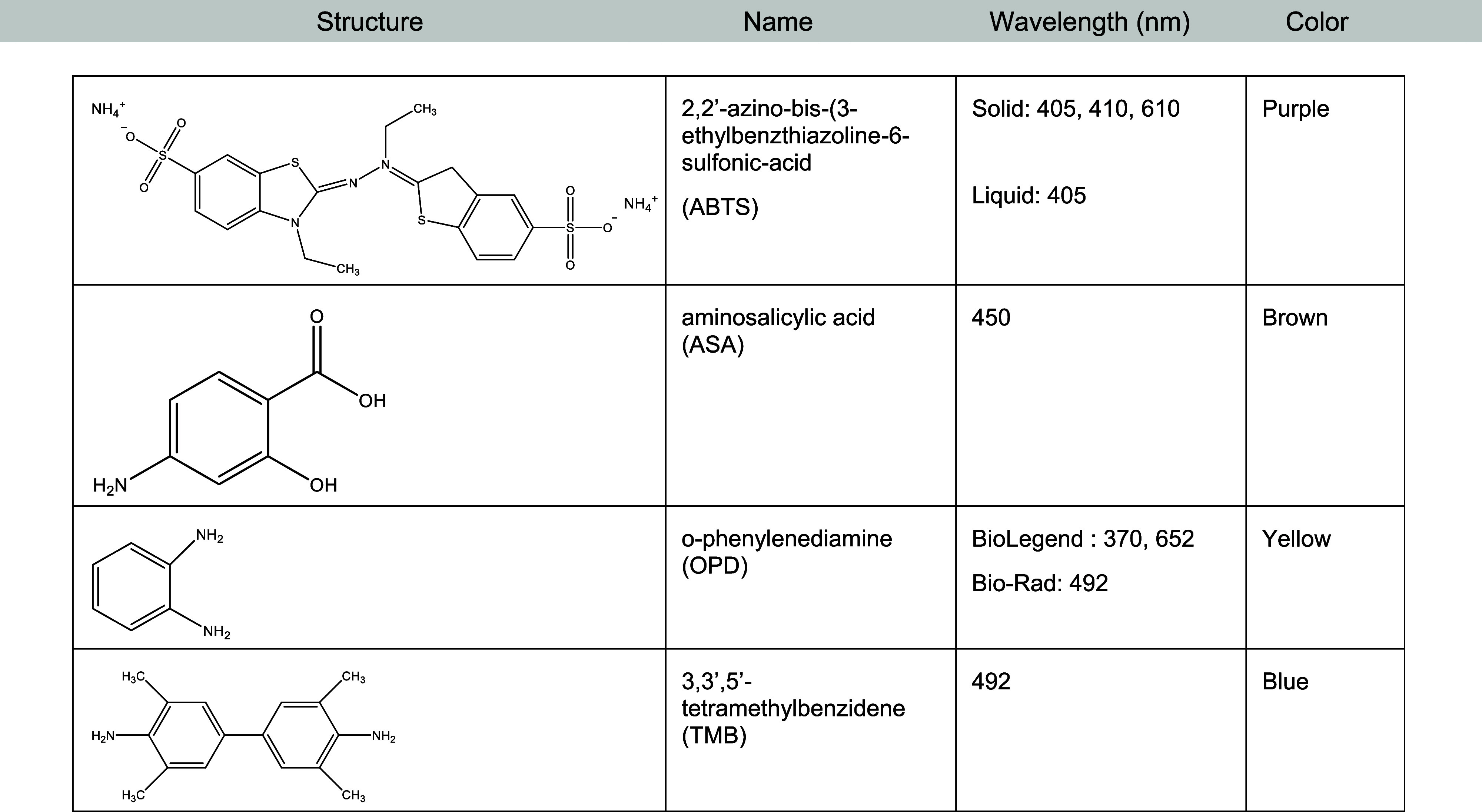
Substrate Data Chart

## Results and Discussion

### Absorbance Measurements

For the next spectrophotometry run, a black 96-well plate was used. After the addition of the substrate solution, there was a yellow color change. After the 30 min incubation time, the solutions maintained the yellow color. After 50 μL of 0.5 M sulfuric acid was added, the yellow color intensified. As the concentration of gluten standards increased, an increase in the intensity of the yellow color should have been present. In addition, there should have been an increase in the absorbance values; however, each measurement was approximately the same. Since the absorbance readings were essentially the same after each run, the gluten standard concentrations were evaluated. Five microliters of each gluten standard was added to 95 μL of bicinchoninic acid (BCA). These solutions were added to a new well plate in duplicate. In addition, duplicate samples of increasing BCA protein concentrations without gluten were run as blanks. The well plate was incubated for 10 min at 37 °C. The BCA assay determines the quantity of protein present in each standard. Prior to being added to the well, the BCA solution is green. As the concentration of protein increases, a purple color is observed. Absorbances were read using a microplate reader at 562 nm. After incubation, each sample remained green without any increase in intensity. In addition, the absorbance for each gluten standard was approximately the same, as previously observed. This indicates that there is not enough protein present in the gluten standards. Therefore, the concentrations of the gluten standards were increased to 0.5, 1.0, 2.0, and 3.0 mg/mL.

Protein determination was repeated by using the BCA assay and more concentrated gluten standards. A purple color gradient was present in the wells, which positively corresponded to the increase in protein concentration. This is evidenced in Table 2. As the concentration increases going down the column, the color intensity visibly increases. Based on the absorbance values, the concentrations of each gluten standard can be calculated and correlated to the theoretical concentration. Table 3 shows the calculated concentrations based on Beer’s law (ε = 2.32 × 10^–1^ L·mol^–1^·cm^–1^). The calculated concentrations were similar to the theoretical concentrations.

**Table 2:** Absorbance Values of More Concentrated Gluten Standards at 562 nm

theoretical concentration	absorbance—trial 1	absorbance—trial 2
0.5 mg/mL	0.117	0.115
1.0 mg/mL	0.125	0.122
2.0 mg/mL	0.247	0.249
3.0 mg/mL	0.264	0.266

**Table 3:** Comparison of Theoretical and Calculated Gluten Standard Concentrations

theoretical concentration (mg/mL)	calculated concentration (mg/mL)	average absorbance
0.5	0.641	0.116
1.0	0.815	0.124
2.0	3.697	0.248
3.0	4.09	0.265

Upon validation of the standards, food samples were tested. Using the BCA assay, pasta samples were used to determine the concentration of gluten in each based on the concentration of gluten standards. Pasta was found to have high levels of gluten. The pasta samples (1 g each) were dissolved in 500 μL of 10% acetic acid separately. Although several of the samples were labeled “gluten-free”, the overall protein content was determined. After obtaining the absorbance of each gluten standard, a plot of the absorbance versus the concentration was prepared, as shown in Figure 2. Using the equation of the straight line and absorbance values from the pasta samples, the concentration of protein present in each sample was calculated. The corresponding absorbance, protein concentration of each pasta sample, and relative ppm levels are presented in Table 4.

**Figure 2:**
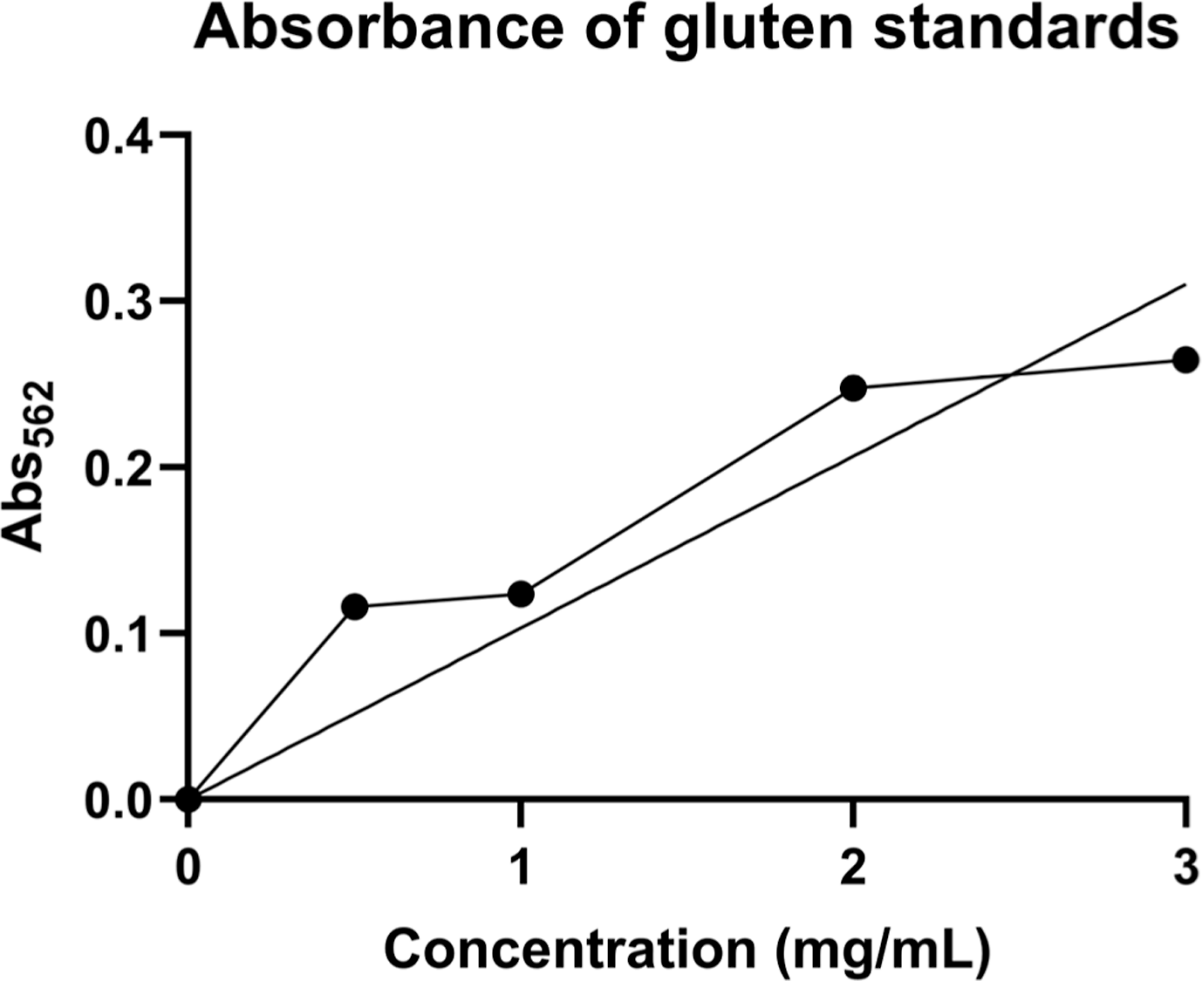
Absorbance versus protein concentration (mg/mL) of gluten standards.

**Table 4:** Measured Absorbance and Calculated Protein in Pasta Samples

pasta brand	absorbance	protein (mg/mL)	ppm
pasta for kids gluten-free	0.714	2.144	2144
oven ready pasta	0.659	1.974	1974
gluten-free penne pasta	0.31	0.898	898
penne gluten-free	0.491	1.454	1454

### Reduced Incubation Times

The original method used for detecting gluten in food samples using an ELISA took approximately 5 h to complete, not including the overnight incubation after the standards and samples were added to the well plate. The purpose of this work is to allow students in a 2.5 h Biochemistry laboratory course to perform an experiment detecting gluten in food products using this biochemical technique. Therefore, each incubation time was cut in half to reduce the total completion time. Following the new procedure, it took approximately 1.5 h to complete, which included the preparation and incubation of the well plate after the standards and samples were added. Based on the standard curve shown in Figure 3, there is still an increasing correlation between gluten concentration and absorbance with reduced incubation times. Therefore, the remainder of this project was completed using the revised protocol.

**Figure 3:**
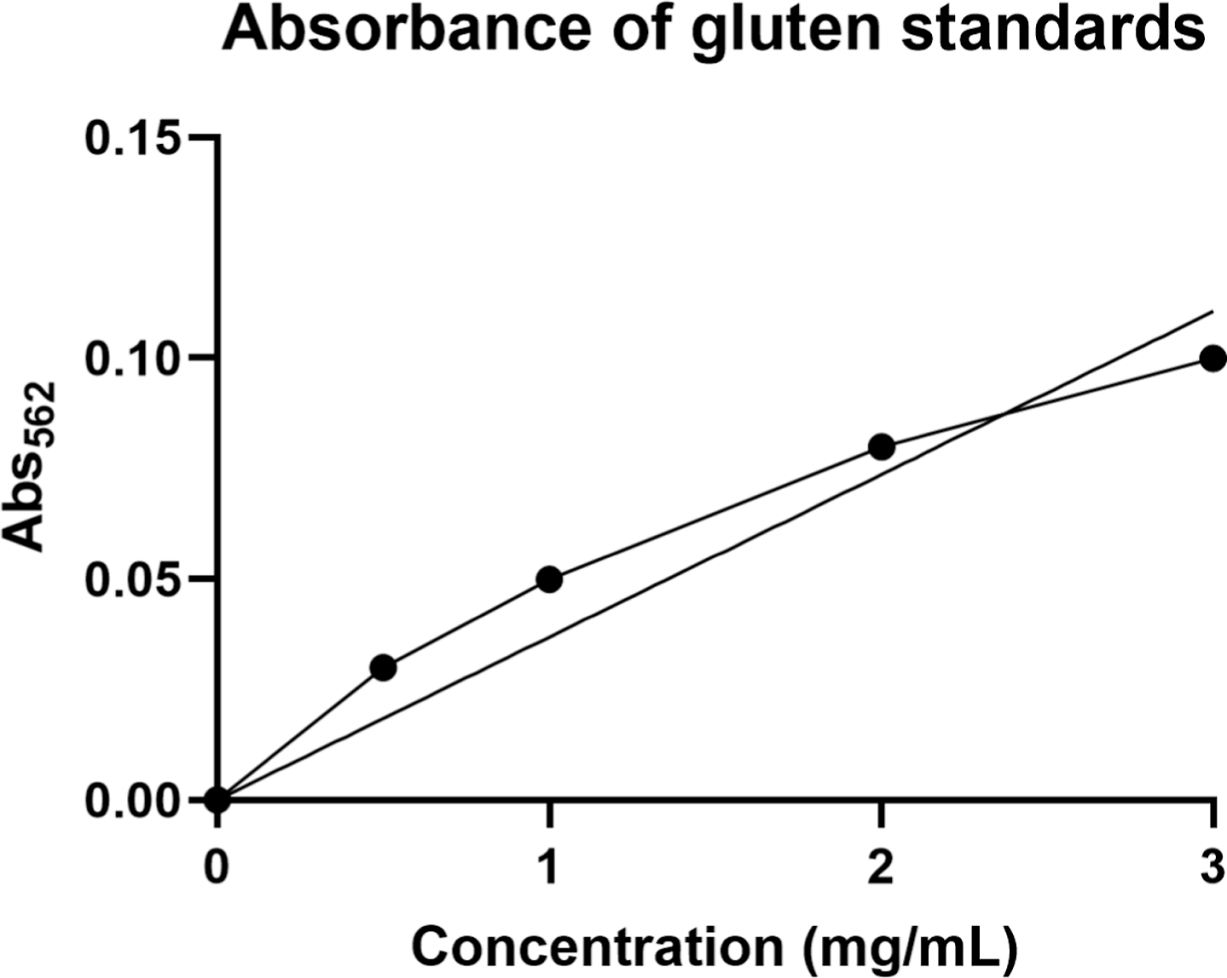
Absorbance versus gluten concentration after reducing incubation times.

### 60% Ethanol Gluten Extraction

The reproducibility of detecting gluten in gluten-containing and gluten-free food products is dependent on the extraction method.^3,5^ Using 60% ethanol ensures that all prolamins are extracted from food samples containing wheat, barley, and rye. Newer extraction methods using chaotropic agents (guanidine hydrochloride) and reducing agents (2-mercaptoethanol) may be better at extracting gluten, but are considered more toxic.^7^ Choline chloride and other deep eutectic solvents may serve as greener alternatives for gluten extraction,^8^ but may not be cost-effective for an undergraduate laboratory for this experiment. As this experiment will be performed by undergraduate students in a traditional laboratory, these reagents are not feasible. In addition, the accuracy of the results is increased by ensuring the antibody is only interacting with the prolamins and no other products are present in the food samples. Table 5 provides a comparison of absorbance measurements obtained with and without gluten extraction with 60% ethanol.

**Table 5:** Comparison of Absorbance Measurements in Food Samples With and Without Ethanol Extraction

food samples	absorbance (extracted with 60% ethanol)	gluten content—ethanol extraction (mg/mL)	absorbance (dissolved in water)	gluten content—water extraction (mg/mL)
all purpose flour	0.118	1.901	0.3235	5.360
rice flour	0.086	1.362	0.2835	4.686
gravy	0.131	2.119	0.2115	3.474
gluten-free gravy	0.089	1.412	0.2085	3.424
whole milk	0.09	1.429	0.1355	2.195
rice milk	0.089	1.412	0.1985	3.256
almond milk	0.077	1.210	0.1865	3.054
beer	0.427	7.103	0.439	7.304

The absorbance measurements are lower with the ethanol extraction, indicating that the extracted gluten is the only antigen interacting with the antibody. Also, it is expected that absorbance values for gluten-free foods would be lower compared with their counterparts. Beer has the highest gluten content, which is expected as it consists of barley and hops. Compared to the milk samples, whole milk (dissolved in ethanol) and rice milk are similar in absorbance values and gluten content.

### Data Analysis

Color gradients and intensities present in gluten standards and food samples were visually observed. Samples with more gluten present were expected to have a more intense color. After, the absorbance was measured for the well plate, and a standard curve was generated for the gluten standards. Using the standard curve, the concentration of gluten present in the food samples was calculated using Beer’s law. The concentration of gluten in the food samples was converted to ppm and compared to the FDA regulation for gluten-free labeling.

### Comparison of Four Different Substrates

The last modification studied was the use of four different substrates that are specifically used to detect the HRP enzyme. Figure 4 shows an image of each well plate, with the color observation corresponding to each substrate. To optimize the absorbance values obtained, the well plate was incubated in 5 min time intervals for a total of 30 min. This allowed for the determination of the best incubation time for each substrate that produced linear absorbance measurements in the gluten standards and higher absorbance measurements in gluten-containing food samples. The first five wells in each well plate correspond to the gluten standards. OPD, BioLegend TMB, and liquid ABTS each have a visible increasing color gradient present. In all samples except those using the ASA and BioRad TMB substrate, gluten-containing food samples have a deeper color than their gluten-free counterparts.

**Figure 4:**
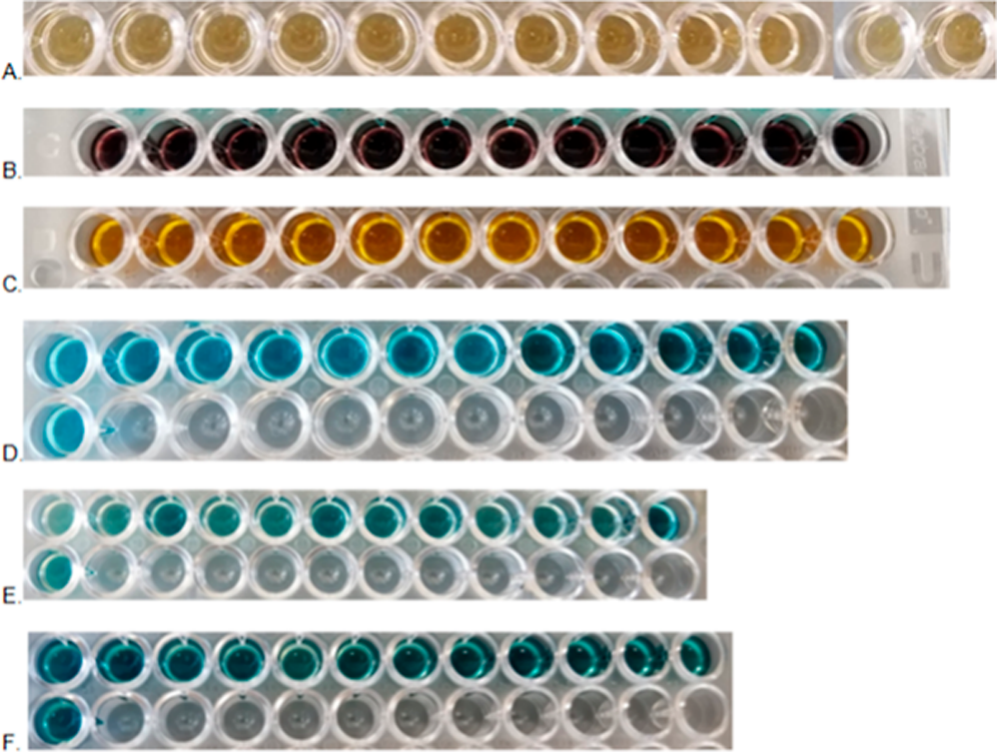
Image of a 96-well plate with 6 different substrates: (A) OPD; (B) ASA; (C)­TMB, BioRad; (D) TMB, BioLegend; (E) ABTS-Liquid; and (F) ABTS-Solid.

Figure 5 shows the gluten standard curve for each substrate. From the direct observation of Figure 5, the OPD produced the best standard curve with an increasing correlation between the absorbance and the concentration. ASA was not plotted as some standard absorbance measurements were out of range, resulting in an “overflow” measurement. Using the other substrates resulted in either fluctuating absorbance measurements or overall decreasing absorbance measurements as the concentration increased.

**Figure 5:**
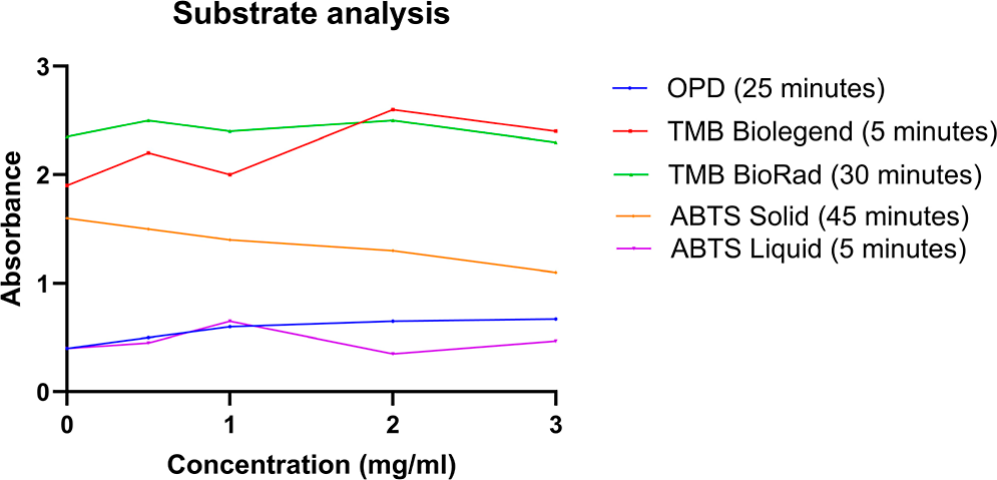
Comparison of five different substrates at different time intervals.

Table 6 shows absorbance measurements obtained for each food sample with the corresponding incubation time for each substrate. It is expected that foods containing gluten should have higher absorbance measurements than their corresponding gluten-free counterpart. For each substrate except ABTS-Solid, both flour and gravy have higher absorbance values than rice flour and gluten-free gravy. In addition, whole milk should have the highest absorbance measurement in the milk samples, as seen in measurements with TMB-BioLegend and ABTS-solid.

**Table 6:** Absorbance Measurements for Each Food Sample With Each Substrat**e**

food samples	OPD	BioLegend	BioRad	ABTS-liquid	ABTS-solid
all-purpose flour	0.674	2.862	2	0.393	1.236
rice flour	0.421	1.763	1.436	0.36	1.164
gravy	0.422	3.02	2.354	0.35	1.269
gluten-free gravy	0.421	2.887	2.294	0.279	1.429
whole milk	0.424	3.078	2.385	0.399	1.345
rice milk	0.431	3.041	2.74	0.383	1.295
almond milk	0.429	3.049	1.92	0.94	1.298
beer	0.427	1.783		0.504	1.451

**Table 7:** Calculated Concentration of Gluten in mg/mL and ppm in Each Food Sample

food samples	concentration (mg/mL)	concentration (ppm)
flour	16.183	16,183
rice flour	3.340	3340
gravy	3.391	3391
gluten-free gravy	3.340	3340
whole milk	3.492	3492
rice milk	3.848	3848
almond milk	3.746	3746
beer	4.254	4254

From the standard curve and the absorbance measurements for the food samples with each substrate, the best substrate(s) can be chosen for future experiments detecting gluten. Based on the data shown, the best substrates are OPD after a 25 min incubation, an incubation of OPD; TMB-BioLegend after 5 min incubation at 492 nm; and last ABTS-liquid after 5 min incubation at 650 nm.

### Accuracy of FDA Regulation Labeling

The overall goal of this work was to quantify the amount of gluten present in gluten-containing and gluten-free foods. More importantly, to determine if gluten-free foods contain up to a maximum of 20 ppm of gluten based on FDA regulations. Using Beer’s law, the amount of gluten in each food sample can be calculated. Table 7 provides the calculated concentration of gluten (mg/mL and ppm) in each food sample using the standard curve using OPD as the substrate after 25 min. All-purpose flour contained the highest amount of gluten with 16,000 ppm on average. Flour is composed of 8–11% gluten, making this result feasible. Flour is a main ingredient present and is used as a thickener when making gravy; therefore, elevated levels of gluten are found in gravy. However, because powdered gravy was used, which has less flour compared to liquid gravy, levels of gluten were lower than expected, as evidenced in Table 7. The second highest concentration is in the beer sample. As gluten is found in wheat and grains, a large component of hops is found in beer; this finding is feasible. Whole milk is considered gluten-free; therefore, the concentration of gluten should be low. The lowest concentration overall was found in the rice flour and gluten-free gravy samples. Rice flour is a high protein alternative that can be used to make processed foods.^9^ Each rice flour sample contains 3340 ppm of gluten on average. However, based on the results, the gluten-free foods evaluated do not meet the FDA regulations and contain significantly higher amounts of gluten.

## Conclusions

This work used a direct ELISA to quantify the amount of antigen (gluten) present in food and beverage samples. Increased concentrations of gluten (0.5, 1.0, 2.0, and 3.0 mg/mL) in standards and absorbance measurements were proportional. In addition, a color gradient was present in the samples. A standard curve for gluten concentration was produced to calculate the amount of gluten in the samples using Beer’s law. All-purpose flour contained the highest concentration of gluten, while gluten-free gravy contained the lowest amount of gluten. This work does have limitations, as the results for samples that are considered gluten-free (rice flour, whole milk, rice milk, almond milk, and gluten-free gravy) should have values that meet the FDA guidelines (20 ppm or less). An affordable antigliadin antibody was used for cost-effectiveness. Upon further review, this antibody is specific to oats, rye, soy, barley, and wheat. This could provide a rationale for the higher than expected values for rice flour and rice milk. If this polyclonal antibody is used in the laboratory, it is recommended that rice and potato food products not be evaluated. The antibody used in this experiment may be best for testing foods that contain oats, barley, rye, soy, and wheat or for qualitative analysis. The R5 and/G12 antibodies may provide more accurate quantitative gluten concentrations in food samples for official testing.^10^ This work has more relevant applications, as new on-site gluten test kits are being validated to ensure precise qualitative results for gluten detection on surfaces and in foods.^11,12^ Reducing the incubation times to ensure that the experiment is completed in a 2.5 h biochemistry laboratory setting had no effect on the results obtained from the assay. To date, over 250 students have completed this experiment. Gluten extraction using ethanol from the food samples resulted in more distinguished absorbance values with gluten-containing and gluten-free foods compared to the previous preparation of dissolving in Millipore water or 10% acetic acid. Of the four substrates, the best correlation was present after a 25 min incubation period with the *o*-phenylenediamine substrate. The best conditions to use when detecting gluten using an ELISA are freshly (<1 h) prepared gluten standards, freshly (<1 h) prepared gluten extracts from food samples using 60% ethanol, and measuring the absorbance of each well after a 25 min incubation period with the OPD substrate. Fresh gluten standards and food samples are imperative in obtaining accurate results for this work. Despite ethanol extraction, absorbance measurements fluctuated in the milk samples. Using standards with higher gluten concentrations is needed as the gluten concentrations in some gluten-free foods were higher than 16 mg/mL. Therefore, it would be best to use gluten standards of 1, 2, 4, 8, 10, 20, and 30 mg/mL in a laboratory experiment. Students in a biochemistry laboratory course will be able to evaluate products containing gluten and those labeled gluten-free to determine if labeling is correct based on FDA regulations in a standard lab session and apply this knowledge to real world experience in making better food choices and sharing that knowledge with others who may have undiagnosed CD or an increased risk based on genetic factors. This experience will enhance their critical thinking skills as well as engage them in extending their fundamental knowledge of biochemistry into real world applications.

## Methods

### Gluten Standards—Original Protocol—Instructor Preparation

Gluten standards with final concentrations of 2, 4, 6, 10, 20, and 30 μg/mL were prepared using 0.05 M sodium carbonate buffer (pH 9.6). A 96-well plate was prepared using 50 μL of each standard and 50 μL of blank sodium carbonate buffer, each in duplicate. All pasta products were dissolved in 500 μL of 10% acetic acid. All other food samples were dissolved in 1 mL of Millipore water (18.2 MΩ).

Gluten was extracted from food samples using 60% ethanol (v/v) to have a final concentration of 1 mg/mL. This extraction method is the standard procedure for gluten extraction and is based on recommendations from the Working Group on Prolamin Analysis and Toxicity.^6^ Samples (1 g) were homogenized for 1 min and then centrifuged for 5 min at 2500*g*. Supernatant was collected. 50 μL of milk samples in duplicate were added to the 96-well plate. Milk samples included fat-free, whole, 1%, soy, goat, almond, and rice milk. The 96-well plate was covered with parafilm and stored overnight at 4 °C. Samples were removed, and each well was washed twice with 100 μL of PBS-T solution. The wells were blocked with a solution of 3% bovine serum albumin (200 μL) and incubated for 1 h at 37 °C. The plate was washed twice with 100 μL of PBS-T. Once washed, 50 μL of freshly prepared gliadin peroxidase conjugated antibody solution (1:1000 in PBS-T with 3% BSA) was added to each well. *New antibody solution must be prepared the day the assay is run.* The plate was covered with aluminum foil and incubated for 1 h at 37 °C. During the incubation, a fresh substrate solution was prepared. The plate was washed twice with 100 μL PBS-T. The appropriate substate (100 μL) was added to each well. The plate was covered with aluminum foil and incubated for 30 min at 37 °C. Sulfuric acid (0.5 M) was used to stop the reaction. Absorbance was measured using a BioTek Instrument Epoch microplate reader (Winooski, VT) at the appropriate wavelength for 30 min at 5 min intervals. The total time for this procedure is ∼ 4.5 h.

### Gluten Standards—Revised Protocol—Instructor Preparation

Gluten standards with final concentrations of 0.5, 1, 2, and 3 mg/mL were prepared using 0.05 M sodium carbonate buffer (pH 9.6). A 96-well plate was prepared using 50 μL of each standard and 50 μL of blank sodium carbonate buffer, each in duplicate. Gluten was extracted from food samples using 60% ethanol to a final concentration of 1 mg/mL. Samples were homogenized for 1 min and then centrifuged for 5 min at 2500g. The supernatant was collected. 50 μL of milk samples in duplicate were added to the 96-well plate. Milk samples included fat-free, whole, 1%, soy, goat, almond, and rice milk. The 96-well plate was covered with parafilm and stored at room temperature for 5 min. The samples were removed, and each well was washed twice with 100 μL of PBS-T solution. The wells were blocked with a solution of 3% bovine serum albumin and incubated for 30 min at 37 °C. The plate was washed twice with 100 μL of PBS-T. Once washed, 50 μL of freshly prepared gliadin peroxidase conjugated antibody solution (1:1000 in PBS-T with 3% BSA) was added to each well. *A new antibody solution must be prepared the day the assay is run.* The plate was covered with aluminum foil and incubated for 5 min at 37 °C. During the incubation, a fresh substrate solution was prepared. The plate was washed twice with 100 μL PBS-T. The appropriate substate (100 μL) was added to each well. The plate was covered with aluminum foil and incubated for 5 min at 37 °C. Using a BioTek Instrument Epoch microplate reader (Winooski, VT), the absorbance of each sample was measured at the appropriate wavelength for 30 min at 5 min intervals. The total time for this procedure is ∼1.5 h.

### Preparation of Substrates—Instructor Preparation

To prepare *o*-phenylenediamine (OPD) substrate, 0.04 g *o*-phenylenediamine (Alfa Aesar, Ward Hills, MA), 30 μL of phosphate citrate buffer, 40 μL of 3% hydrogen peroxide (Fisher Science Education, Hanover Park, IL), and Millipore water were added to reach 10 mL total. The solution was then vortexed. Two different types of 3,3′,5,5′-tetramethylbenzidiene (TMB) substrates, BioLegend and BioRad, were used. Two milliliters of BioLegend TMB Substrate Reagent A and 2 mL of BioLegend TMB Substrate Reagent B were combined and vortexed. The BioRad HRP Enzyme Substrate was used as manufactured. To prepare aminosalicylic acid (ASA) substrate, 0.034 g of aminosalicylic acid (Alfa Aesar, Wood Hill, MA), 13.5 mL of PBS-T, and 1.5 mL of 3% hydrogen peroxide (Fisher Science Education, Hanover Park, IL) were combined and vortexed. Two different states, liquid and solid, of 2,2′-azino-bis­(3-ethylbenzthiazoline-6-sulfonic-acid) diammonium salt (ABTS) substrate were tested. The Invitrogen Super AquaBlue ELISA Substrate (Thermo Fisher Scientific, San Diego, CA) was used as manufactured. The solid was prepared by adding 10 mg of ABTS solid (Alfa Aesar, Wood Hill, MA) to a solution of phosphate citrate buffer (pH 5.0) and 10 μL 30% hydrogen peroxide (Fisher Chemicals, Fair Lawn, NJ). The solution was vortexed before using.
